# Prevalence of skin injuries related to medical adhesives in Brazilian hospitals: a multicenter study

**DOI:** 10.1590/0034-7167-2024-0279

**Published:** 2025-12-08

**Authors:** Débora Ferreira Pio, Samuel de Paula Pinheiro da Silva, Daniel Nogueira Cortez, Juliano Teixeira Moraes

**Affiliations:** I Universidade Federal de São João del-Rei. Divinópolis, Minas Gerais, Brazil

**Keywords:** Adhesives, Wounds and Injuries, Nursing Care, Intensive Care Unit, Prevalence., Adhesivos, Heridas y Lesiones, Cuidado de Enfermera, Unidad de Terapia Intensiva, Predominio.

## Abstract

**Objectives:**

to analyze the overall prevalence of skin injuries related to medical adhesives (MARSI) in patients admitted to adult intensive care units.

**Methods:**

this was a cross-sectional, multicenter study conducted in four hospitals across three Brazilian states. Data analysis was performed using descriptive statistics, inferential analysis, and logistic regression.

**Results:**

a total of 108 patients participated, with a MARSI prevalence of 25.9%. Among the identified types, maceration was the most common (13 cases; 39.4%), followed by irritant contact dermatitis (8 cases; 24.2%). A statistically significant association was found with the global subjective nutritional assessment (*p* = 0.032), peripheral venous access fixation (*p* = 0.030), and the number of adhesives used (*p* = 0.001).

**Conclusions:**

the prevalence of MARSI was 25.9%, with significant associations with the global subjective nutritional assessment, peripheral venous access fixation, and the number of adhesives used.

## RESUMEN

**Objetivos:**

analizar la prevalencia global de lesiones cutáneas relacionadas con adhesivos médicos (MARSI) en pacientes ingresados en unidades de cuidados intensivos para adultos.

**Métodos:**

estudio transversal y multicéntrico realizado en cuatro hospitales de tres estados brasileños. El análisis de los datos se llevó a cabo mediante estadística descriptiva, inferencial y regresión logística.

**Resultados:**

participaron 108 pacientes, con una prevalencia de MARSI del 25,9%. Entre los tipos identificados, la maceración fue la más común (13 casos; 39,4%), seguida de la dermatitis de contacto irritativa (8 casos; 24,2%). Se observó una asociación estadísticamente significativa con la evaluación subjetiva del estado nutricional (*p* = 0,032), la fijación del acceso venoso periférico (*p* = 0,030) y el número de adhesivos utilizados (*p* = 0,001).

**Conclusiones:**

la prevalencia de MARSI fue del 25,9%, estando asociada con la evaluación subjetiva del estado nutricional, la fijación del acceso venoso periférico y la cantidad de adhesivos utilizados.

## INTRODUCTION

Clinical care in hospital settings often requires the use of medical devices in various regions of the body. According to the World Health Organization, a medical device is defined as any instrument, apparatus, equipment, machine, implant, reagent for in vitro use, software, material, or other similar article intended by the manufacturer for human use, either alone or in combination, with a specific medical purpose^(1)^.

Many of these devices are secured to the skin using adhesives. When used in patient care, these materials are referred to as medical adhesives and are found in tapes, dressings, fixation devices, and electrodes. The risk of skin damage caused by medical adhesives is already widely recognized in the scientific literature^(2)^. However, their inadvertent application or removal can result in skin injuries^(3,4)^.

Skin injuries resulting from the use of medical adhesives are internationally referred to as Medical Adhesive-Related Skin Injury (MARSI). This condition is characterized by the appearance of erythema and/or other skin alterations, such as vesicles, blisters, erosions, or tears that persist for 30 minutes or more after adhesive removal^(2)^. MARSI compromises epidermal integrity, causes pain, and increases the risk of infection, negatively affecting the quality of care provided^(5)^. Several factors may contribute to its occurrence. Intrinsic factors include age, race, ethnicity, underlying diseases (such as diabetes mellitus, infections, malnutrition, dehydration), and dermatological conditions (such as eczema or dermatitis). Extrinsic factors include repeated application and removal of adhesives at the same site, prolonged exposure to moisture or skin dryness, as well as treatments such as radiation and certain medications^(2,4,5)^.

Because they are often perceived as routine occurrences and not as adverse events, MARSI cases are underreported in Brazil. However, this is a preventable condition with a direct impact on care costs and patient safety^(4,6)^. With the advancement of knowledge on the subject, studies have reported the occurrence of MARSI. Nevertheless, significant gaps remain regarding its incidence and prevalence.

An incidence rate of 31% was identified among critically ill adult and elderly oncology patients admitted to an intensive care unit (ICU), associated with the use of adhesives for peripheral venous catheter fixation in a Brazilian capital city. In another cohort study conducted in two university hospitals in Brazil, an incidence of 42% was observed^(7)^. In China, a study in a university hospital with 430 patients identified an incidence of 11.86%^(8)^. Another cohort in China, conducted in 2024, reported an incidence of 33% associated with the use of adhesives for peripherally inserted central catheter (PICC) fixation^(9)^.

Given this scenario, there is a clear need for further investigations to better understand the scope of MARSI and its associated factors. Such knowledge is essential to raise awareness among healthcare professionals and managers about the importance of implementing effective prevention strategies. Proper management of adhesives can significantly reduce hospital costs, patient pain, and the length of hospital stay resulting from avoidable complications.

## OBJECTIVES

To analyze the prevalence of Medical Adhesive-Related Skin Injury (MARSI) and associated factors in patients admitted to adult intensive care units in Brazilian hospitals.

## METHODS

### Ethical aspects

The project was submitted to the Research Ethics Committee of the coordinating center and the respective research centers, receiving favorable approval for its execution in all evaluations.

The Informed Consent Form (ICF) was signed by the participant and, in cases where decision-making was not possible (due to sedation or clinical condition), the invitation to participate was extended to the legally responsible family member.

This article is derived from the master’s thesis entitled “Prevalence of Skin Injuries Related to the Use of Medical Adhesives in Brazilian Hospitals: A Multicenter Study”, defended in 2021 in the Graduate Program in Nursing at the Federal University of São João del-Rei^(10)^.

### Design

This was a cross-sectional study guided by the STROBE tool (*Strengthening the Reporting of Observational Studies in Epidemiology*)^(11)^.

### Period

Data collection was conducted from November to December 2020.

### Setting

The research was carried out in four Brazilian research centers, all large hospitals considered reference institutions for public and/or private healthcare, each with an adult ICU, located in three Brazilian states. These included: a philanthropic hospital in the city of São Paulo (State of São Paulo - SP) with 36 ICU beds; a private hospital in Brasília (Federal District - DF) with 53 ICU beds; a philanthropic hospital in Divinópolis (State of Minas Gerais - MG1) with 40 ICU beds; and a philanthropic hospital in Belo Horizonte (State of Minas Gerais - MG2) with 30 ICU beds.

### Population and sample

All adult patients admitted to general ICU beds on the day of data collection participated in the study. Ten patients were excluded because they were not in the unit at the time of the physical examination or were undergoing surgical procedures. The final population consisted of 108 patients: 27 from the SP unit, 17 from the DF unit, 49 from the MG1 unit, and 15 from the MG2 unit.

### Inclusion and exclusion criteria

Participants aged 18 years or older, with at least one type of adhesive applied to any region of the body, were included in the study. Patients who could not undergo a physical examination at the time of assessment due to hemodynamic instability and those who were not present in the unit due to diagnostic or surgical procedures were excluded.

### Study variables

The categorical variable was the presence or absence of a skin injury related to the use of medical adhesives, as classified by the Consensus Statements for the Assessment, Prevention, and Treatment of Adhesive-Related Skin Injurie*s* ^(2,4)^. This document presents the following classification: mechanical trauma injury (skin stripping, tension or blister injury, friction injury); dermatitis-type injury (irritant contact dermatitis, allergic dermatitis); and others (maceration and folliculitis).

The independent variables included sociodemographic and clinical data: age in years; race (White and non-White); global subjective assessment (nourished, malnourished); skin condition (moisture, texture, turgor, edema); comorbidities (diabetes, hypertension); medications used on the day (antibiotic, anticoagulant, electrolytes, diuretics, corticosteroids, vasodilator); other treatments; number of adhesives used; types of adhesives used (nasogastric/nasoenteric catheter fixation, central venous access fixation, peripheral venous access fixation); adhesive location (chest, upper limbs); backing type (foam, porous); adhesive type (film, acrylic, rubber); and the occurrence of skin injury related to the adhesive.

### Data collection

Data were collected exclusively by the four researchers responsible for each research center, all of whom received theoretical and practical training on skin physical examination, the concept and identification of MARSI types, and the characterization of the adhesive materials used.

### Research instrument

Two instruments were used to record the information. The first instrument included a section for identifying the research center, date, number, and period of data collection. Subsequently, the sociodemographic and clinical characteristics of the participants were recorded, including: “patient’s initials, bed number, age, sex, race, global subjective assessment”^(12)^, skin condition (moisture, texture, turgor, edema), reasons for hospitalization (ICD-10), comorbidities, medications in use on the day, and other treatments. During this stage, the independent variables were recorded, obtained from the medical records and the participant’s clinical and skin assessment. Skin inspection was performed in a cephalocaudal direction, with attention to regions where one or more types of adhesives were present, and the dependent variable (presence of injury) was observed.

The second instrument referred to variables related to skin injury associated with medical adhesives, such as: number of adhesives; presence of an institutional protocol for MARSI prevention; device type (nasogastric/nasoenteric catheter fixation, nasal catheter fixation, central venous access fixation, peripheral venous access fixation, PICC fixation, electrodes, ostomy equipment, dressing, surgical dressing, urinary catheter fixation); adhesive location (cranium/occipital, face, cervical, anterior chest, posterior chest, abdomen, back, sacral, upper limbs - ULs, lower limbs - LLs); adhesive materials: backing (not applicable, paper, fabric, porous, polyurethane film, foam) and adhesive type (not applicable, silicone, rubber, acrylic, elastomeric polymer - hydrocolloid). Regarding the occurrence of MARSI, the classification proposed by McNichol^(3)^ was used: none; mechanical injury - friction injury; mechanical injury - adhesive removal and reapplication; mechanical injury due to tension or adhesive blister; allergic contact dermatitis; irritant contact dermatitis; maceration; folliculitis.

The instruments were developed by a Brazilian research and study group in stomatherapy and were subsequently tested and adapted at the research centers prior to execution.

### Data processing and analysis

Data were analyzed using the Statistical Package for the Social Sciences (SPSS), version 21.0, through descriptive statistics and bivariate analyses, including Pearson’s chi-square test, Fisher’s exact test, and the Mann-Whitney test. The measure of association was the odds ratio, and logistic regression was used for multivariate analysis. The statistical significance level adopted was 5% (*p*-value < 0.05).

## RESULTS

The population consisted of 108 participants admitted to ICU, with 49 (45.4%) in MG1, 27 (25.0%) in SP, 17 (15.7%) in DF, and 15 (13.9%) in MG2. Half of the facilities had a specific institutional protocol for MARSI prevention. In total, 33 participants with MARSI were identified, corresponding to a prevalence of 25.9% in the ICU. The MG1 center had the highest prevalence at 30.6%, followed by DF with 29.4%, MG2 with 20.0%, and SP with 14.8%.

Regarding sociodemographic and clinical data, the mean age was 62.3 years (SD = 17.50), ranging from 23 to 98 years. There was a predominance of males, with 58 participants (56.3%), while 45 (43.7%) were female.

The main reasons for hospitalization among the participants were: ICD Z95 (Presence of cardiac and vascular implants and grafts) and ICD Z98 (Other postprocedural states), corresponding to 4.6% of the sample; and ICD C50 (Malignant neoplasm of the breast), ICD I64 (Stroke, not specified as hemorrhagic or ischemic), and ICD R57 (Shock, not elsewhere classified), each accounting for 3.7%.

At the time of data collection, participants were using medications such as antibiotics (62.0%), anticoagulants (51.9%), electrolyte replacements (39.8%), and diuretics (32.4%). Some were also undergoing other adjunct clinical treatments, such as immunosuppressants (1.9%), chemotherapy, and radiotherapy (0.9%) (Table 1).

**Table 1:** Association between the presence and absence of Medical Adhesive-Related Skin Injury and demographic and clinical aspects in patients admitted to Brazilian intensive care units during the period of November/December 2020, Brazil (N=108)

Variables	Without MARSI n (%)	With MARSI n (%)	*p* value
Sociodemographic variables			
Age			
> 60 years	44 (74.6)	15 (25.4)	0.911^1^
≤ 60 years	37 (75.5)	12 (24.5)	
Skin color			
White	50 (69.4)	22 (30.6)	0.081^1^
Non-white	25 (86.2)	4 (13.8)	
Global Subjective Assessment			
Nourished	48 (72.7)	18 (27.3)	0.032^*2^
Malnourished	13 (100.0)	0 (0.0)	
Clinical variables			
Comorbidities			
Diabetes	18 (78.3)	5 (21.7)	0.747^1^
Arterial hypertension	41 (80.4)	10 (19.6)	0.280^1^
Main medications in use			
Antibiotic	50 (74.6)	17 (25.4)	0.909^1^
Anticoagulant	42 (74.6)	14 (25.4)	0.657^1^
Electrolytes	33 (76.4)	10 (23.3)	0.734^1^
Diuretic	24 (68.6)	11 (31.4)	0.285^1^
Corticosteroids	12 (75.0)	4 (25.0)	1.000^2^
Vasodilator	9 (90.0)	1 (10.0)	0.445^2^
Skin condition - Moisture			
Normal	67 (74.4)	23 (25.6)	1.000^2^
Dry/Sweaty	14 (77.8)	4 (22.2)	
Skin condition - Texture			
Thin	68 (75.6)	22 (24.4)	0.761^2^
Eutrophic	12 (70.6)	5 (29.4)	
Skin condition - Turgor			
Normal	58 (73.4)	21 (26.6)	0.590^1^
Decreased	22 (78.6)	6 (21.4)	
Skin condition - Edema			
With edema	31 (73.8)	11 (26.2)	0.708^2^
Without edema	7 (63.6)	4 (36.4)	

Significant p value;

1 Pearson’s chi-square test;

2 Fisher’s exact test; MARSI: Medical Adhesive-Related Skin Injury.

*Note: For the sociodemographic variables (Table 1), the global subjective assessment (p = 0.032) was significantly associated with the occurrence of skin injury related to medical adhesives.*

No statistically significant associations were identified between the categorical clinical variables and the occurrence of injury. On average, each participant used six adhesives, with a minimum of two and a maximum of 14 adhesives (SD = 2.28). When testing the statistical difference between individuals who used a higher or lower number of adhesives and the presence of MARSI, it was found that the greater the number of adhesives, the higher the likelihood of developing MARSI (*p* = 0.001). As these were patients admitted to the ICU, it is characteristic that they carried more than one medical adhesive. The most frequently used devices were electrodes (98.1%), peripheral venous access fixation (62.0%), and central venous access fixation (46.3%). The main locations were the anterior chest region (37.9%) and the upper limbs (24.9%).

A statistically significant association was observed between MARSI and the use of peripheral venous access fixation (*p* = 0.030). The type of adhesive or its backing did not show a significant difference in MARSI prevalence in this population (Table 2).

**Table 2:** Association between the presence and absence of Medical Adhesive-Related Skin Injury and adhesives/location in patients admitted to Brazilian intensive care units during the period of November/December 2020, Brazil (N=108)

Variables	Without MARSI n (%)	With MARSI n (%)	*p* value
Adhesives in use			
Nasogastric/nasoenteric catheter fixation	17 (63.0)	10 (37.0)	0.095^1^
Central venous access fixation	38 (76.0)	12 (24.0)	0.824^1^
Peripheral venous access fixation	55 (82.1)	12 (17.9)	0.030^*1^
Location			
Chest	78 (74.3)	27 (25.7)	0.571^2^
Upper limbs	62 (77.5)	18 (22.5)	0.310^1^
Adhesive backing			
Foam	80 (74.8)	27 (25.2)	1.000^2^
Porous	48 (72.7)	18 (27.3)	0.494^1^
Type of adhesive			
Film	38 (77.6)	11 (22.4)	0.577^1^
Acrylic	66 (76.7)	20 (23.3)	0.408^1^
Rubber	59 (79.7)	15 (20.3)	0.094^1^

Significant p value;

1 Pearson’s chi-square test;

2 Fisher’s exact test; MARSI: Medical Adhesive-Related Skin Injury.

Maceration-type MARSI and dermatitis were the most frequently identified, corresponding to 39.4% and 45.4% of the injuries, respectively. Among contact dermatitis cases, the occurrence of the irritant type was 24.2% (Table 3).

**Table 3:** Occurrence of skin injuries related to medical adhesives in hospitalized patients (N=108) in intensive care units, Brazil, 2020

Type of MARSI	n (%)
Skin tear (mechanical)	01 (3.0)
Skin stripping (mechanical)	03 (9.1)
Tension injury (mechanical)	01 (3.0)
Allergic contact dermatitis	07 (21.2)
Irritant contact dermatitis	08 (24.2)
Maceration (others)	13 (39.4)
Folliculitis (others)	0.0 (0.0)
Amount	33 (100.0)

## DISCUSSION

Studies on skin injuries related to medical adhesives (MARSI) are essential, especially given the scarcity of epidemiological investigations on the subject. The prevalence of these injuries is a key indicator of care quality, as MARSI constitutes a preventable adverse event that remains underrecognized by healthcare professionals and is often neglected in the Brazilian clinical context^(13)^.

This study demonstrated a high prevalence in Brazilian ICU, consistent with findings from other studies. Among them, a multicenter study with critically ill patients evaluated 377 individuals, of whom 61 presented with MARSI, resulting in an overall prevalence of 16.2%^(14)^. In China, a cross-sectional study conducted in a pediatric ICU followed 232 participants over two weeks, revealing a MARSI prevalence ranging from 23.53% to 54.17%, with an average of 37.15%^(15)^.

In Brazil, a cohort study conducted in the state of Minas Gerais followed 83 participants in a cardiac ICU for up to 15 days after admission, reporting a MARSI incidence of 25.3%^(16)^. The sample characterization data and factors associated with skin injuries related to medical adhesives indicated no significant association with age group. Although the literature suggests that such injuries are more common in patients with fragile skin-such as premature neonates and the elderly-considered more vulnerable to adhesive tape use, this physiological predisposition has not been confirmed in epidemiological studies conducted in ICU^(2,4,17,18)^.

Although no association with age was observed, extrinsic factors may be related to the occurrence of MARSI. These include dry skin resulting from the use of aggressive cleansing products, excessive bathing, low air humidity, prolonged exposure to moisture, certain medications, radiotherapy, adhesive removal and application, sun exposure, and frequent changes of dressings and catheter fixation tapes. Such conditions are common among critically ill patients^(19,20)^.

Regarding skin color, individuals who self-identified as non-White showed a lower chance of developing MARSI (an estimated reduction of 265%). Additionally, the use of adhesives on the upper limbs was also associated with a lower probability of injury occurrence (a 72% reduction).

This study also identified an association between the global subjective nutritional assessment and the occurrence of MARSI. The literature indicates that nutritional impairment can affect skin integrity and hinder wound healing^(20)^. Although malnourished patients have a physiological predisposition, in this study, most injuries were identified in patients classified as nourished, suggesting that any individual using medical adhesives is potentially at risk of developing MARSI, as also indicated by international consensus statements^(2)^.

Although skin assessment parameters such as moisture, texture, turgor, and the presence of edema did not show statistical significance, they may influence MARSI occurrence. In ICUs, it is common for patients to present skin changes due to hemodynamic instability and the continuous use of medical devices-factors that promote skin fragility^(21,22)^.

Regarding the presence of comorbidities, such as hypertension and diabetes, no associations with MARSI were observed. The same applies to the use of medications such as antibiotics, anticoagulants, electrolytes, diuretics, corticosteroids, and vasodilators. Nevertheless, studies suggest that the chronic use of certain drugs, such as corticosteroids, may contribute to the development of these injuries^(23)^.

Another relevant finding of this study was the relationship between the number of adhesives used and the increased likelihood of developing MARSI. The greater the number of adhesive devices, the higher the skin exposure and, consequently, the greater the risk of injury. This finding is supported by another study conducted in a cardiac ICU^(16)^. The most commonly used adhesives among participants were those with a foam backing and an acrylic adhesive surface. Regarding the adhesive location, a high percentage was observed in the chest region and upper limbs. There was also significant use of devices such as electrodes and peripheral venous access fixation with microporous adhesive. Other studies have reported similar data: electrodes and peripheral venous access fixation were the most frequently used products during data collection^(16,24)^. This usage is directly related to the characteristics of the intensive care environment, such as the application of occlusive adhesives for prolonged periods and inadequate removal techniques-factors not measured in this investigation^(2)^.

The type of device also showed a statistically significant association with MARSI, especially in the case of peripheral venous access fixation. A similar result was observed in a study with 697 patients, which found a MARSI prevalence of 19.7% in patients using PICCs^(25)^. Other studies indicate that patients with vascular devices often require daily adhesive changes, which increases the risk of injury^(26)^. These findings may be explained by a Brazilian prevalence study conducted in a cardiac ICU, which identified dressing change frequency as a risk factor for MARSI. According to this study, during the first 24 hours after catheter insertion, polyurethane film and gauze dressings are used; subsequently, only the film is used, as per institutional protocol, provided the insertion site remains clean and dry^(27)^.

Maceration was the most frequently observed type of MARSI. According to McNichol^(4)^, maceration presents as wrinkled, whitish, or grayish skin, resulting from epidermal softening and increased permeability, making the skin more susceptible to friction damage or irritating agents. This occurs partly due to the loss of the barrier function of the stratum corneum, which is responsible for preventing excessive water loss. Transepidermal water loss (TEWL), widely used as an indicator of barrier function effectiveness, quantifies the amount of water lost through the skin^(28)^.

Thus, prolonged contact with moisture-resulting from bathing, sweating, or wet adhesives-favors the onset of these injuries. The maceration observed in this study was also related to the frequent use of microporous adhesives and electrodes, although, in other studies, this type of MARSI was not the most prevalent. Skin care is fundamental in preventing injuries, including protection against mechanical or chemical damage. The use of medical adhesives is common in healthcare institutions, and it is rare for a patient not to undergo interventions involving adhesive use, which predisposes the patient to adhesive-related injuries^(2,4,29)^. Skin injuries in general have received special attention from healthcare professionals due to their socioeconomic impact on healthcare institutions and the negative aspect associated with the quality of care provided to patients.

A strength of this study was the participation of four Brazilian centers in different states, providing a representative sample that allowed for larger case series (megatrials). The study used a research instrument previously validated in the pilot study, and the data analysis technique addressed the clustered nature of the data and controlled for possible confounding factors. Data collection was supervised by the research coordinators, and the researchers were previously trained, thereby increasing data reliability.

### Study limitations

Among the limitations of this study, it is important to note that, being a multicenter study, there was no uniformity in medical record documentation, as each institution had specific particularities that were not investigated in this study. Therefore, information regarding comorbidities and other adjunct treatments depended on the data recorded in the medical charts, which, at the time of data collection, may not have contained all relevant information. These limitations are consistent with other cross-sectional studies, in which data are collected only once and provide a snapshot in time.

### Contributions to the field

This study contributes to the understanding of the epidemiological behavior of skin injuries related to medical adhesives in ICU, promoting improvements in the quality of care.

## CONCLUSIONS

The study results indicated a 25.9% prevalence of skin injuries related to medical adhesives, with maceration being the most frequent subtype. The global subjective nutritional assessment and peripheral venous access fixation were factors associated with the occurrence of these injuries. Additionally, it was observed that the risk of injury increased with the number of adhesives used.

## Data Availability

The research data are available only upon request.
