# Supplementary material for: Prevalence of skin injuries related to medical adhesives in Brazilian hospitals: a multicenter study
Source: Rev Bras Enferm. 2025 Dec 8;78(Suppl 4):e20240279. doi: 10.1590/0034-7167-2024-0279 (PMC12700576; doi:10.1590/0034-7167-2024-0279)
Supplement: Translation

# Translation: Prevalência de lesões de pele relacionadas a adesivos médicos em hospitais brasileiros: estudo multicêntrico

**DOI:** 10.1590/0034-7167-2024-0279pt

**Authors:** Débora Ferreira Pio, Samuel de Paula Pinheiro da Silva, Daniel Nogueira Cortez, Juliano Teixeira Moraes

**Affiliations:** I Universidade Federal de São João del-Rei. Divinópolis, Minas Gerais, Brasil

**Keywords:** Adesivos, Ferimentos e Lesões, Cuidados de Enfermagem, Unidade de Terapia Intensiva, Prevalência.

## Abstract

**Objetivos:**

analisar a prevalência global de lesões de pele relacionadas a adesivos médicos (MARSI) em pacientes internados em unidades de terapia intensiva adulta.

**Métodos:**

estudo transversal e multicêntrico realizado em quatro hospitais de três estados brasileiros. A análise dos dados foi conduzida por meio de estatística descritiva, inferencial e regressão logística.

**Resultados:**

participaram 108 pacientes, com uma prevalência de 25,9% de MARSI. Dentre os tipos identificados, a maceração foi a mais comum (13 casos; 39,4%), seguida da dermatite de contato irritativa (8 casos; 24,2%). Houve associação estatisticamente significativa para a avaliação subjetiva do estado nutricional (p = 0,032), a fixação do acesso venoso periférico (p = 0,030) e o número de adesivos utilizados (p = 0,001).

**Conclusões:**

a prevalência de MARSI foi de 25,9%, estando associada à avaliação subjetiva do estado nutricional, à fixação do acesso venoso periférico e à quantidade de adesivos utilizados.

## INTRODUÇÃO

Os cuidados clínicos em ambientes hospitalares frequentemente demandam o uso de dispositivos médicos em diversas regiões do corpo. Segundo a Organização Mundial da Saúde, dispositivo médico é definido como qualquer instrumento, aparelho, equipamento, máquina, implante, reagente para uso *in vitro, software*, material ou outro artigo semelhante, destinado pelo fabricante para uso humano, isoladamente ou em combinação, com finalidade médica específica^(1)^.

Muitos desses dispositivos são fixados à pele por meio de adesivos. Quando empregados no cuidado ao paciente, esses materiais são denominados adesivos médicos, sendo encontrados em fitas, curativos, dispositivos de fixação e eletrodos. O risco de danos cutâneos causados por adesivos médicos já é amplamente reconhecido na literatura científica^(2)^. No entanto, sua aplicação ou remoção inadvertida pode resultar em lesões na pele^(3,4)^.

As lesões decorrentes do uso de adesivos médicos são internacionalmente denominadas *Medical Adhesive-Related Skin Injury* (MARSI). Essa condição é caracterizada pelo aparecimento de eritema e/ou outras alterações cutâneas, como vesículas, bolhas, erosões ou rupturas, que persistem por 30 minutos ou mais após a remoção do adesivo^(2)^. As lesões MARSI comprometem a integridade epidérmica, provocam dor e aumentam o risco de infecção, afetando negativamente a qualidade da assistência prestada^(5)^. Diversos fatores podem contribuir para sua ocorrência. Entre os intrínsecos, destacam-se idade, raça, etnia, doenças subjacentes (como diabetes mellitus, infecções, desnutrição, desidratação) e condições dermatológicas (como eczema ou dermatite). Já os fatores extrínsecos incluem a aplicação e remoção repetida de adesivos no mesmo local, exposição prolongada à umidade ou ao ressecamento da pele, bem como o uso de tratamentos como radiação e determinados medicamentos^(2,4,5)^.

Por serem frequentemente encaradas como situações rotineiras e não como eventos adversos, as lesões MARSI são subnotificadas no Brasil. No entanto, trata-se de um agravo evitável, com impacto direto nos custos assistenciais e na segurança do paciente^(4,6)^. Com o avanço do conhecimento sobre o tema, estudos vêm relatando a ocorrência de MARSI. Ainda assim, há lacunas importantes quanto à sua incidência e prevalência.

Uma taxa de incidência de 31% foi identificada em pacientes adultos e idosos oncológicos críticos, internados em unidade de terapia intensiva (UTI), associada ao uso de adesivos para fixação de cateter venoso periférico, em uma capital brasileira. Em outro estudo de coorte realizado em dois hospitais universitários no Brasil, foi observada incidência de 42%^(7)^. Na China, estudo em hospital universitário com 430 pacientes identificou a incidência de 11,86%^(8)^. Outra coorte na China, em 2024, identificou a incidência de 33% pelo uso de adesivo em cateter venoso central de inserção periférica (^(9)^.

Diante desse cenário, é evidente a necessidade de mais investigações que permitam compreender melhor a dimensão da MARSI e seus fatores relacionados. Tal conhecimento é essencial para sensibilizar profissionais e gestores da saúde quanto à importância de implementar estratégias eficazes de prevenção. O manejo adequado dos adesivos pode reduzir significativamente os custos hospitalares, a dor dos pacientes e o tempo de internação decorrente de complicações evitáveis.

## OBJETIVOS

Analisar a prevalência de *Medical Adhesive-Related Skin Injury* (MARSI) e fatores associados em pacientes internados em unidades de terapia intensiva adulta de hospitais brasileiros.

## MÉTODOS

### Aspectos éticos

O projeto foi submetido ao Comitê de Ética em Pesquisa do centro coordenador e dos respectivos centros de pesquisa, tendo parecer favorável para sua execução em todas as apreciações.

O Termo de Consentimento Livre e Esclarecido foi assinado pelo participante e, na impossibilidade de tomada de decisão (sedação ou condição clínica), o convite foi realizado ao familiar responsável.

Este artigo é derivado da dissertação de mestrado intitulada “Prevalência de lesões de pele relacionadas ao uso de adesivos médicos em hospitais brasileiros: um estudo multicêntrico”, defendida em 2021 no Programa de Pós-Graduação em Enfermagem da Universidade Federal de São João del-Rei^(10)^.

### Desenho

Trata-se de um estudo transversal, norteado pela ferramenta *STROBE* (*Strengthening the Reporting of Observational Studies in Epidemiology*)^(11)^.

### Período

A coleta de dados ocorreu nos meses de novembro a dezembro do ano de 2020.

### Local

A pesquisa foi realizada em quatro centros de pesquisa brasileiros, hospitais de grande porte, referência para a assistência pública e/ou privada, dotados de UTI adulta, distribuídos em três estados brasileiros. Um hospital filantrópico na cidade de São Paulo (Estado - São Paulo - SP), com 36 leitos de UTI; um hospital privado em Brasília (Estado - Distrito Federal - DF), com 53 leitos de UTI; um hospital filantrópico em Divinópolis (Estado - Minas Gerais - MG1), com 40 leitos de UTI; e um hospital filantrópico em Belo Horizonte (Estado - Minas Gerais - MG2), com 30 leitos de UTI.

### População e amostra

Participaram da pesquisa todos os pacientes adultos internados em leitos de UTI geral no dia da coleta. Dez pacientes foram excluídos por não estarem na unidade no momento do exame físico ou por se encontrarem em procedimentos cirúrgicos. A população foi composta por 108 pacientes, sendo 27 pacientes da unidade SP, 17 pacientes da unidade do DF, 49 da unidade MG1 e 15 pacientes da unidade MG2.

### Critérios de inclusão e exclusão

Foram incluídos, na pesquisa, participantes maiores de 18 anos de idade, com, no mínimo, um tipo de adesivo em qualquer região do corpo. Foram excluídos aqueles com impossibilidade de realização do exame físico no momento da avaliação, em razão da instabilidade hemodinâmica, e participantes que não se encontravam na unidade, em decorrência de procedimentos diagnósticos ou cirúrgicos.

### Variáveis do estudo

A variável categórica foi a presença ou ausência da lesão relacionada ao uso de adesivos médicos, conforme classificação estabelecida pelo consenso *Consensus Statements for the Assessment, Prevention, and Treatment of Adhesive-Related Skin Injuries* ^(2,4)^. Nesse documento, apresenta-se a seguinte classificação: Lesão por trauma mecânico: desnudamento da pele, lesão por tensão ou bolha, lesão por fricção; lesão do tipo dermatite: dermatite irritativa de contato, dermatite alérgica; outras: maceração e foliculite.

As variáveis independentes referem-se aos dados sociodemográficos e clínicos: idade em anos; raça (branco e não branco); avaliação subjetiva global (nutrido, desnutrido); condição da pele (umidade, textura, turgor, edema); comorbidades (diabetes, hipertensão arterial); medicamentos em uso no dia (antibiótico, anticoagulante, eletrólitos, diuréticos, corticosteroides, vasodilatador); outros tratamentos; número de adesivos utilizados; adesivos usados (fixação de cateter nasoentérico/nasogástrico, fixação de acesso venoso central, fixação de acesso venoso periférico); localização do adesivo (tórax, membros superiores); tipo de dorso (espuma, poroso); tipo de adesivo (filme, acrílico, borracha) e ocorrência da lesão de pele relacionada ao adesivo.

### Coleta de dados

Os dados foram coletados exclusivamente pelos quatro pesquisadores responsáveis por cada centro de pesquisa, que receberam treinamento teórico-prático sobre exame físico da pele, conceito e identificação dos tipos de MARSI e caracterização do insumo adesivo utilizado.

### Instrumento de pesquisa

Foram utilizados dois instrumentos para o registro das informações. O primeiro instrumento era composto por uma etapa de identificação do centro de pesquisa, data, número e período da coleta dos dados. Subsequentemente, foram identificadas as características sociodemográficas e clínicas dos participantes, com: “iniciais do nome do paciente, número do leito, idade, sexo, raça, avaliação subjetiva global”^(12)^, condição da pele (umidade, textura, turgor, edema), motivos de internação CID-10, comorbidades, medicamentos em uso no dia e outros tratamentos. Durante essa etapa, foram registradas as variáveis independentes, obtidas a partir de informações do prontuário e da avaliação clínica e da pele do participante. A inspeção da pele foi realizada no sentido céfalo-podálico, com atenção para regiões que apresentavam um ou mais tipos de adesivos, sendo observada a variável dependente (presença de lesão).

O segundo instrumento refere-se às variáveis relacionadas à lesão de pele associada a adesivos médicos, como: número de adesivos; existência de protocolo institucional para a prevenção de MARSI; tipo de dispositivo (fixação de cateter nasogástrico/entérico, fixação de cateter nasal, fixação de acesso venoso central, fixação de acesso venoso periférico, fixação de cateter central de inserção periférica (PICC), eletrodos, equipamentos de estomias, curativo, curativo cirúrgico, fixação de cateter urinário); localização do adesivo (crânio/occipital, face, cervical, tórax anterior, tórax posterior, abdome, dorso, sacral, membros superiores - MMSS, membros inferiores - MMII); insumos adesivos: dorso (não se aplica, papel, tecido, poroso, filme de poliuretano, espuma) e tipo de adesivo (não se aplica, silicone, borracha, acrílico, polímero elastomérico - hidrocolóide). Quanto à ocorrência de MARSI, utilizou-se a classificação proposta por McNichol^(3)^: não possui; lesão mecânica - lesão por fricção; lesão mecânica - remoção e reaplicação do adesivo; lesão mecânica por tensão ou bolha do adesivo; dermatite de contato alérgica; dermatite de contato irritativa; maceração; foliculite.

Os instrumentos foram desenvolvidos por um núcleo de estudos e pesquisa em estomaterapia brasileiro e, posteriormente, foram realizados testes e adequações nos centros de pesquisa antes da execução.

### Tratamento e análise dos dados

Os dados foram analisados pelo *Statistical Package for the Social Sciences* (SPSS), versão 21.0, por meio de estatística descritiva e análises bivariadas, com teste qui-quadrado de Pearson, teste exato de Fisher e teste de Mann-Whitney. A medida de associação foi *odds ratio* (OR) e foi utilizada a regressão logística como análise multivariada. O nível de significância estatística adotado foi de 5% (*valor-p* < 0,05).

## RESULTADOS

A população foi composta por 108 participantes internados em unidades de terapia intensiva, sendo 49 (45,4%) em MG1, 27 (25%) em SP, 17 (15,7%) no DF e 15 (13,9%) em MG2. Metade dos serviços possuía protocolo institucional específico para a prevenção de MARSI. No total, foram identificados 33 participantes com MARSI, correspondendo a uma prevalência de 25,9% nas unidades de terapia intensiva. O centro MG1 apresentou a maior prevalência, com 30,6%, enquanto o DF registrou uma prevalência de 29,4%, MG2 20% e SP 14,8%. Em relação aos dados sociodemográficos e clínicos, registrou-se idade média de 62,3 anos (DP = 17,50), variando de 23 a 98 anos. Houve predomínio do sexo masculino, com 58 participantes (56,3%), enquanto 45 (43,7%) eram do sexo feminino.

Os principais motivos da internação dos participantes foram: Classificação Internacional das Doenças (CID) Z95 (Presença de implantes e enxertos cardíacos e vasculares) e CID Z98 (Outros estados pós-cirúrgicos), correspondendo a 4,6% da amostra; e CID C50 (Neoplasia maligna da mama), CID I64 (Acidente vascular cerebral, não especificado como hemorrágico ou isquêmico) e CID R57 (Choque não classificado em outra parte), correspondendo a 3,7%.

No momento da coleta de dados, os participantes faziam uso de medicamentos do tipo antibiótico (62%), anticoagulantes (51,9%), reposição de eletrólitos (39,8%) e diuréticos (32,4%). Alguns também realizavam outros tratamentos clínicos adjuvantes, como, por exemplo, uso de imunossupressores (1,9%), quimioterapia e radioterapia (0,9%) (Tabela 1).

**Tabela 1:** Associação entre presença e ausência de *Medical Adhesive-Related Skin Injury* e aspectos demográficos e clínicos em pacientes internados em unidades de terapia intensiva brasileiras no período de novembro/dezembro de 2020, Brasil (N=108)

Variáveis	Sem MARSI n (%)	Com MARSI n (%)	Valor de *p*
Variáveis sociodemográficas			
Idade			
> 60 anos	44 (74,6)	15 (25,4)	0,911^1^
≤ 60 anos	37 (75,5)	12 (24,5)	
Cor da pele			
Branca	50 (69,4)	22 (30,6)	0,081^1^
Não branca	25 (86,2)	4 (13,8)	
Avaliação Subjetiva Global			
Nutrido	48 (72,7)	18 (27,3)	0,032^*2^
Desnutrido	13 (100,0)	0 (0,0)	
Variáveis clínicas			
Comorbidades			
Diabetes	18 (78,3)	5 (21,7)	0,747^1^
Hipertensão arterial	41 (80,4)	10 (19,6)	0,280^1^
Principais medicamentos em uso			
Antibiótico	50 (74,6)	17 (25,4)	0,909^1^
Anticoagulante	42 (74,6)	14 (25,4)	0,657^1^
Eletrólitos	33 (76,4)	10 (23,3)	0,734^1^
Diurético	24 (68,6)	11 (31,4)	0,285^1^
Corticosteroides	12 (75,0)	4 (25,0)	1,000^2^
Vasodilatador	9 (90,0)	1 (10,0)	0,445^2^
Condição da pele - Umidade			
Normal	67 (74,4)	23 (25,6)	1,000^2^
Seca/Sudorenta	14 (77,8)	4 (22,2)	
Condição da pele - Textura			
Fina	68 (75,6)	22 (24,4)	0,761^2^
Eutrófica	12 (70,6)	5 (29,4)	
Condição da pele - Turgor			
Normal	58 (73,4)	21 (26,6)	0,590^1^
Diminuído	22 (78,6)	6 (21,4)	
Condição da pele - Edema			
Com edema	31 (73,8)	11 (26,2)	0,708^2^
Sem edema	7 (63,6)	4 (36,4)	

Valor de p significativo;

1 Teste qui-quadrado de Pearson;

2 Teste exato de Fisher; MARSI - Medical Adhesive-Related Skin Injury.

*Obs.: Para as variáveis sociodemográficas (Tabela 1), a avaliação subjetiva global (p = 0,032) associou-se significativamente com a ocorrência de lesão de pele relacionada a adesivos médicos.*

Não foram identificadas associações estatisticamente significativas entre as variáveis clínicas categóricas e a ocorrência de lesão.

Em média, cada participante utilizava seis adesivos, com uso mínimo de dois e máximo de 14 adesivos (DP = 2,28). Ao testar a diferença estatística entre as pessoas que usavam maior ou menor número de adesivos e a presença de MARSI, evidenciou-se que, quanto maior o número de adesivos, maior a chance de desenvolver MARSI (*p* = 0,001). Como se trata de pacientes internados na UTI, é característico que esses portem mais de um adesivo médico. Os dispositivos mais utilizados foram os eletrodos (98,1%), a fixação de acesso venoso periférico (62%) e a fixação de acesso venoso central (46,3%). A principal localização foi a região do tórax anterior (37,9%) e os MMSS (24,9%).

Observou-se uma representatividade estatística na associação de MARSI com o uso da fixação do acesso venoso periférico (p = 0,030). O tipo de adesivo ou seu dorso não apresentou diferença significativa para a prevalência de MARSI nesta população (Tabela 2).

**Tabela 2:** Associação entre presença e ausência de *Medical Adhesive-Related Skin Injury* e adesivos/localização em pacientes internados nas unidades de terapia intensiva brasileiras no período de novembro/dezembro de 2020, Brasil (N=108)

Variáveis	Sem MARSI n (%)	Com MARSI n (%)	Valor de *p*
Adesivos em uso			
Fixação Cateter nasogástrico/entérico	17 (63,0)	10 (37,0)	0,095^1^
Fixação Acesso Venoso Central	38 (76,0)	12 (24,0)	0,824^1^
Fixação Acesso Venoso Periférico	55 (82,1)	12 (17,9)	0,030^*1^
Localização			
Tórax	78 (74,3)	27 (25,7)	0,571^2^
Membros superiores	62 (77,5)	18 (22,5)	0,310^1^
Dorso do Adesivo			
Espuma	80 (74,8)	27 (25,2)	1,000^2^
Poroso	48 (72,7)	18 (27,3)	0,494^1^
Tipo de adesivo			
Filme	38 (77,6)	11 (22,4)	0,577^1^
Acrílico	66 (76,7)	20 (23,3)	0,408^1^
Borracha	59 (79,7)	15 (20,3)	0,094^1^

Valor de p significativo;

1 Teste qui-quadrado de Pearson;

2 Teste exato de Fisher; MARSI - Medical Adhesive-Related Skin Injury.

A MARSI do tipo maceração e dermatites foi a mais identificada, correspondendo a 39,4% e 45,4% das lesões, respectivamente. Dentre as dermatites de contato, a ocorrência do tipo irritativa foi de 24,2% (Tabela 3).

**Tabela 3:** Ocorrência das lesões de pele relacionadas a adesivos médicos em pacientes internados (N=108) nas unidades de terapia intensiva, Brasil, 2020

Tipos de MARSI	n (%)
Lesão por Fricção (mecânica)	01 (3,0)
Desnudamento da pele (mecânica)	03 (9,1)
Lesão por tensão (mecânica)	01 (3,0)
Dermatite alérgica (dermatites)	07 (21,2)
Dermatite de contato irritativa(dermatites)	08 (24,2)
Maceração (outras)	13 (39,4)
Foliculite (outras)	0,0 (0,0)
Total	33 (100,0)

## DISCUSSÃO

Estudos sobre lesões de pele relacionadas a adesivos médicos (MARSI) são imprescindíveis, especialmente diante da escassez de investigações epidemiológicas sobre o tema. A prevalência dessas lesões é um indicador essencial da qualidade da assistência, uma vez que a MARSI constitui um evento adverso prevenível, ainda pouco reconhecido pelos profissionais de saúde e frequentemente negligenciado no contexto clínico brasileiro^(13)^.

Este estudo demonstrou uma prevalência expressiva nas unidades de terapia intensiva brasileiras, assim como em outros estudos. Dentre eles, em um estudo multicêntrico com pacientes críticos, dos 377 pacientes avaliados, 61 apresentaram MARSI, obtendo-se uma prevalência global de 16,2%^(14)^. Na China, um estudo transversal realizado em uma terapia intensiva pediátrica acompanhou 232 participantes ao longo de duas semanas, revelando uma prevalência de MARSI que variou de 23,53% a 54,17% (média de 37,15%)^(15)^.

Já no Brasil, uma coorte realizada no estado de Minas Gerais acompanhou 83 participantes em uma UTI cardiológica por até 15 dias após a admissão, encontrando uma incidência de MARSI de 25,3%^(16)^. Os dados de caracterização da amostra e os fatores associados às lesões de pele relacionadas ao uso de adesivos médicos indicaram que não houve associação significativa com a faixa etária. Embora a literatura aponte que tais lesões são mais comuns em pacientes com pele frágil - como neonatos prematuros e idosos -, considerados mais vulneráveis ao uso de fitas adesivas, essa predisposição fisiológica não tem sido confirmada em estudos epidemiológicos realizados em unidades de terapia intensiva^(2,4,17,18)^.

Embora não se observe associação com a idade, fatores extrínsecos podem estar relacionados à ocorrência de MARSI. Entre eles, destacam-se: pele seca, decorrente do uso de produtos de limpeza agressivos, banhos excessivos, baixa umidade do ar, exposição prolongada à umidade, medicamentos, casos de radioterapia, remoção e aplicação do adesivo, exposição solar e troca frequente de curativos e fitas de fixação de cateteres. Tais condições são comuns em pacientes críticos^(19,20)^.

Quanto à cor da pele, observou-se que indivíduos que se autodeclaram de cor diferente da branca apresentaram menor chance de desenvolver MARSI (redução estimada em 265%). Além disso, o uso de adesivos na região dos MMSS também esteve associado a uma menor probabilidade de ocorrência da lesão (redução de 72%).

Este estudo também identificou associação entre a avaliação subjetiva do estado nutricional e a ocorrência de MARSI. A literatura indica que o comprometimento nutricional pode afetar a integridade da pele e dificultar a cicatrização de lesões^(20)^. Embora pacientes desnutridos apresentem predisposição fisiológica, neste estudo, a maioria das lesões foi identificada em pacientes classificados como nutridos, o que sugere que qualquer indivíduo em uso de adesivo médico está potencialmente sujeito a desenvolver MARSI, como também apontado por consensos internacionais^(2)^.

Ainda que os parâmetros de avaliação da pele, como umidade, textura, turgor e presença de edema, não tenham apresentado significância estatística, eles podem influenciar na ocorrência de MARSI. Em UTIs, é comum que pacientes apresentem alterações cutâneas em decorrência da instabilidade hemodinâmica e do uso contínuo de dispositivos médicos, fatores que favorecem a fragilidade da pele^(21,22)^.

No que se refere à existência de comorbidades, como hipertensão e diabetes, não foram observadas associações com MARSI. O mesmo se aplica ao uso de medicamentos como antibióticos, anticoagulantes, eletrólitos, diuréticos, corticosteroides e vasodilatadores. Ainda assim, estudos sugerem que o uso crônico de alguns fármacos, como corticosteroides, pode contribuir para o desenvolvimento dessas lesões^(23)^.

Outro achado relevante deste estudo foi a relação entre o número de adesivos utilizados e a maior chance de desenvolver MARSI. Quanto maior a quantidade de dispositivos adesivos, maior a exposição cutânea e, portanto, o risco de lesões. Este achado é corroborado por outro estudo realizado em UTI cardiológica^(16)^. Os adesivos mais utilizados pelos participantes foram aqueles com dorso de espuma e, na parte adesiva, de acrílico. Quanto à localização do adesivo, foi demonstrado um percentual elevado na região do tórax e MMSS. Também foi expressivo o uso de dispositivos como eletrodos e a fixação de acesso venoso periférico com adesivo microporoso. Estudos apontam alguns dados semelhantes: os eletrodos e a fixação de acesso venoso periférico foram os produtos mais utilizados durante a coleta de dados^(16,24)^. Esse uso está diretamente relacionado às características do ambiente intensivo, como a aplicação de adesivos oclusivos por longos períodos e técnicas inadequadas de remoção - aspectos não mensurados nesta investigação^(2)^.

O tipo de dispositivo também apresentou associação estatisticamente significativa com MARSI, especialmente no caso da fixação do acesso venoso periférico. Resultado semelhante foi observado em estudo com 697 pacientes, no qual se encontrou uma prevalência de MARSI de 19,7% em pacientes com a utilização de PICC^(25)^. Outros estudos apontam que pacientes com dispositivos vasculares frequentemente necessitam de trocas diárias dos adesivos, o que aumenta o risco de lesão^(26)^. Esses achados podem ser justificados por estudo brasileiro de prevalência em UTI cardiológica, que apontou a frequência de troca dos curativos como fator de risco para MARSI. Segundo esse estudo, as primeiras 24 horas após a inserção do cateter envolvem o uso de coberturas com filme de poliuretano e gaze; posteriormente, utiliza-se apenas o filme, conforme protocolo institucional, desde que a inserção esteja limpa e seca^(27)^.

A maceração foi o tipo de MARSI mais frequentemente observado. De acordo com McNichol^(4)^, a maceração se manifesta por pele enrugada, esbranquiçada ou acinzentada, resultado do amolecimento da epiderme e do aumento da permeabilidade, o que a torna mais suscetível a danos por fricção ou agentes irritantes. Isso ocorre, em parte, devido à perda da função de barreira do estrato córneo, responsável por impedir a perda excessiva de água. A perda transepidérmica de água (*TEWL*), amplamente usada como indicador da eficácia dessa barreira, quantifica a água que se perde pela pele^(28)^.

Assim, o contato prolongado com umidade - proveniente do banho, sudorese ou adesivos molhados - favorece o surgimento dessas lesões. A maceração observada neste estudo também se relaciona com o uso frequente de adesivos microporosos e eletrodos, embora, em outras pesquisas, esse tipo de *MARSI* não tenha sido o mais prevalente. O cuidado da pele é fundamental na prevenção de lesões, inclusive na proteção contra lesões mecânicas ou químicas. O uso de adesivo médico é comum nas instituições de saúde, sendo raro um paciente não receber intervenções que envolvam o uso de adesivos, o que predispõe o paciente a lesões causadas por adesivos^(2,4,29)^. As lesões de pele, em geral, têm recebido destaque especial dos profissionais de saúde, em decorrência do impacto socioeconômico para as instituições de saúde e do aspecto negativo que envolve a qualidade da assistência prestada ao paciente.

O ponto forte deste estudo foi a participação de quatro centros brasileiros, em estados diferentes, tendo uma amostra representativa que permitiu a obtenção de casuísticas maiores (*megatrials*). O estudo utilizou um instrumento de pesquisa validado previamente no estudo piloto, e a técnica de análise de dados abordou a natureza agrupada dos dados e controlou os possíveis fatores de confusão. A coleta de dados foi supervisionada pelos coordenadores da pesquisa, e os pesquisadores foram previamente treinados, aumentando assim a confiabilidade dos dados.

### Limitações do estudo

Dentre as limitações do estudo, registra-se o fato de que, por ser um estudo multicêntrico, não havia uniformidade nos registros de informações no prontuário, uma vez que cada instituição possui particularidades específicas que não foram investigadas neste estudo. Assim, informações sobre comorbidades e outros tratamentos adjuvantes dependiam dos registros no prontuário, que, naquele momento da coleta, poderiam não conter todas as informações. Tais limitações corroboram com outros estudos transversais, nos quais os dados são coletados apenas uma vez e fornecem um instantâneo no tempo.

### Contribuições para a área

O estudo contribui para o conhecimento do comportamento epidemiológico da lesão de pele relacionada ao adesivo médico em unidades de terapia intensiva, favorecendo melhorias na qualidade da assistência.

## CONCLUSÕES

Os resultados do estudo indicaram uma prevalência de 25,9% de lesões de pele relacionadas a adesivos médicos, com a maceração como subtipo mais frequente. A avaliação subjetiva do estado nutricional e a fixação do acesso venoso periférico foram fatores associados à ocorrência das lesões. Além disso, observou-se que o risco de lesão aumenta conforme o número de adesivos utilizados.

## Data Availability

Os dados de pesquisa só estão disponíveis mediante solicitação.
